# Examining the integration of artificial intelligence in supply chain management from Industry 4.0 to 6.0: a systematic literature review

**DOI:** 10.3389/frai.2024.1477044

**Published:** 2025-01-20

**Authors:** Alexander Samuels

**Affiliations:** Department of Transport Economics and Logistics Management, North West, South Africa

**Keywords:** artificial intelligence, human-centric integration, Industry 4.0, Industry 5.0, Industry 6.0, supply chain management, sustainability

## Abstract

**Background:**

This study examines the integration of Artificial Intelligence (AI) in supply chain management (SCM) during the transition from Industry 4.0 to Industry 6.0. The focus is on improving operational efficiency, promoting human-centric collaboration, and advancing sustainability within supply chains. As industries progress, the need to incorporate AI technologies that improve decision-making and operational resilience while ensuring sustainable practices becomes increasingly critical. This systematic review aims to explore how AI is transforming SCM through these industrial transitions.

**Methods:**

Utilising the PRISMA framework, a systematic review was conducted to gather and analyse relevant literature published between 2010 and 2023. A comprehensive search of databases including Web of Science, Scopus, IEEE Xplore, Google Scholar, and ScienceDirect was performed. The review involved rigorous screening for eligibility and thematic analysis using Atlas-ti software to identify key themes and patterns related to AI integration in SCM.

**Results:**

The findings indicate that AI integration significantly improves SCM by improving demand forecasting, inventory management, and overall decision-making capabilities. Industry 5.0 focuses on human-AI collaboration, improving customisation and problem-solving. AI technologies also contribute to sustainability by optimising resource utilisation and reducing environmental impacts. However, challenges such as cybersecurity risks and workforce skill gaps need to be addressed to fully leverage AI’s potential.

**Conclusion:**

Integrating AI in SCM not only improves operational efficiency and sustainability but also promotes resilience against disruptions. The insights from this review offer valuable guidance for both academics and practitioners aiming to optimise supply chain operations through AI technologies from Industry 4.0 to Industry 6.0. The study underlines the importance of a balanced approach that integrates technological developments with human-centric and sustainable practices.

## 1 Introduction

Over the years, supply chain management has experienced a significant transformation, with an emphasis on the management of resource flows to generate and distribute value to end consumers (Mukhamedjanova, 2020). Businesses seeking to improve their competitiveness, efficiency, and ability to adapt to market changes have recognised the importance of incorporating supply chain management principles (Fu, 2023). Enterprises, particularly those of small and medium size, are utilising supply chain management strategies to obtain competitive advantages and improve corporate performance (Gong, 2023). The core of supply chain management is the integration of key business operations to improve value for customers and stakeholders (Mukhamedjanova, 2020).

Furthermore, the concept of Green Supply Chain Management (GSCM) has recently been introduced, which involves the integration of environmental considerations into supply chain practices to increase flexibility in adhering to environmental regulations, reduce waste, and improve social responsibility (Cao, 2024). This development in supply chain management entails the synchronisation of supply, production, and sales procedures to efficiently improve the capabilities of a firm and minimise expenses (Dou, 2022). Additionally, the progress in Artificial Intelligence (AI) and machine learning is transforming supply chain management through improvements in demand forecasting, routing, and scheduling (Petriashvili, 2024).

Eyo-Udo (2024) notes that the quality, cost, and reliability of supply chains are being significantly influenced by the increasing application of AI technologies in supplier selection and management. AI is transforming supply chain management by optimising procedures, increasing decision-making, and improving overall efficiency (Chukwu, 2024). AI integration in supply chains is regarded as a solution for tackling difficulties such as precise prediction, efficient inventory control, and maintaining transparency across the supply chain (Singh et al., 2023).

The integration of Artificial Intelligence (AI) technologies has become more critical in the transformation of supply chain management from Industry 4.0 to Industry 6.0. The integration of artificial intelligence (AI) into supply chains has various advantages, including the improvement of efficiency, agility, responsiveness, and competitiveness (Joel, 2024). AI-powered innovation is key for improving the resilience and performance of supply chains by tackling previously neglected aspects (Belhadi et al., 2021).

Through the utilisation of AI technology, organisations are able to streamline activities, predict patterns, improve efficiency, and encourage creativity, resulting in a significant transformation of logistics and supply chain management (Richey, 2023). The integration of AI into supply chain management is key for organisations to adjust to the changing demands of the market and technological developments (Samuels et al., 2024b). AI technologies, like as robotics and automation, have transformed warehouse and distribution operations, improved operational efficiency and encouraged strategic innovation (Muthaluri, 2024). However, AI-powered predictive analytics are key in predicting demand, optimising supply, and reducing disruptions, hence improving the efficiency and resilience of the supply chain (Elufioye, 2024).

Integrating human resources with AI and Machine Learning (ML) technologies is key for achieving a unified supply chain management system, improving flexibility, adaptability, and competitiveness in the current dynamic business landscape. Industry 4.0 established the groundwork for sophisticated AI applications in supply chains by introducing major technological pillars, including cybersecurity, IoT, big data, and robotics (Trevisan and Formentini, 2024). As we go towards Industry 6.0, the integration of AI technology continues to transform supply chain operations by improving decision-making, decreasing expenses, and optimising resource distribution (Eyo-Udo, 2024). Comprehending the advantages and difficulties linked with the transition from Industry 4.0 to Industry 6.0 demands an analysis of the development and influence of AI technologies on supply chain management. The utilisation of AI and sensor technology has demonstrated the ability to improve animal welfare standards, diminish supply chain errors, and augment operational productivity across diverse industries (Neethirajan, 2023). The integration of artificial intelligence (AI) into supply chains provides advantages such as improved punctuality in delivery, increased efficiency in labour, and decreased carbon dioxide (CO_2_) emissions per unit produced (Silva, 2024).

Recognising the potential of AI-driven predictive analytics to optimise supply chains by forecasting demand, managing inventory, and improving overall response to market changes is a key step in identifying the main advantages and difficulties in integrating AI within supply chains (Nzeako, 2024). Nevertheless, in order to guarantee the effective integration of AI technologies in supply chain management, it is imperative to tackle challenges such as cybersecurity risks highlighted by Kumar and Mallipeddi (2022) and the requirement for sustainable practices emphasised by Samuels et al. (2024b). Strategic recommendations for the future integration of AI in supply chains from Industry 4.0 to Industry 6.0 require the cultivation of a culture that prioritises sustainability and cybersecurity, the investment in technology sharing, and the establishment of comprehensive regulatory frameworks (Layode, 2024). Furthermore, to effectively implement circular supply chain processes in Industry 6.0, organisations should prioritise collaboration, investments in new technology, and talent development (Sami et al., 2023). Drawing from the background of this study, the research objectives are:

- To analyse the evolution and impact of AI technologies on supply chain management from Industry 4.0 to Industry 6.0
- To identify key benefits and challenges in the Integration of AI within supply chains
- To provide strategic recommendations for future integration of AI in supply chains from Industry 4.0 to Industry 6.0

The transition from Industry 4.0 to Industry 6.0 marks a significant evolution in the industrial and technological landscape, emphasising not only automation and digitalisation but also the integration of advanced artificial intelligence (AI) technologies, human-centric approaches, and improved sustainability. This study’s significance lies in its comprehensive examination of this transition, particularly focusing on the integration of AI in supply chain management.

## 2 Literature review

### 2.1 Overview on AI in supply chain management

Recent literature has devoted considerable attention to the integration of Artificial Intelligence (AI) into supply chain management. Research has emphasised the favourable influence of artificial intelligence (AI) technology on supply chain operations, specifically highlighting improvements in operational efficiency, strategic innovation, and sustainability (Samuels et al., 2024a). AI applications have demonstrated the ability to improve the agility, flexibility, and resilience of supply chains and organisations, especially during disruptive events such as the COVID-19 pandemic (Siagian et al., 2021). AI-driven predictive analytics are key for optimising supply chains as they improve operational efficiencies, decrease costs, and improve customer satisfaction (Nzeako, 2024).

AI’s importance grows as supply networks transition from Industry 4.0 to Industry 6.0. AI technologies, including Artificial Neural Networks (ANN), Genetic Algorithms (GA), Virtual Reality (VR), and Artificial Immune Systems (AIS), are being used to improve the performance of supply chain management (Mohsen, 2023). The integration of AI and ML with human resources is proposed to establish a mutually beneficial relationship that utilises technology and human creativity for the purpose of integrated supply chain management (Samuels et al., 2024a). Challenges persist in the implementation of AI in supply chains, despite the potential advantages. The literature is currently addressing issues such as cybersecurity, sustainability, and the need for improved decision-making processes (Layode, 2024). The strategic recommendations for the future integration of AI in supply chains highlight the need to tackle these challenges and utilise AI-driven innovation to improve operational efficiency, customer satisfaction, and gain a competitive edge.

### 2.2 Evolution of AI technologies from Industry 4.0 to Industry 6.0

The development of AI technologies from Industry 4.0 to Industry 6.0 has had a significant impact on supply chain management, providing transformative benefits and presenting distinctive challenges. AI technologies, including machine learning, predictive analytics, and optimisation algorithms, have allowed organisations to improve the resilience of their supply chains, increase efficiency, lower costs, and improve overall performance (Samuels et al., 2024c).

The progress in technology has transformed the way supply chain operations are conducted, resulting in increased levels of effectiveness, adaptability, and competitiveness in the current dynamic corporate environment (Elufioye, 2024). It has been demonstrated that supply chain management using AI may reduce disruptions, adjust to changing market conditions, allocate resources optimally, and improve operational effectiveness (Eyo-Udo, 2024). Through the utilisation of AI technologies, businesses could automate processes, predict trends, customise experiences, optimise operations, reduce risks, and encourage creativity (Joel, 2024). Additionally, AI-powered systems have significantly changed the way warehousing and distribution operations are conducted, demonstrating the immense potential of AI to completely overhaul supply chain activities (Muthaluri, 2024). AI technologies, such as machine learning and big data analytics, have enabled progress in the analysis of real-time data, predictive maintenance, and resource optimisation in agricultural and IT industry supply chains (Elufioye, 2024). These technologies have played a key role in improving the security, efficiency, and ability to recover of supply chains. They have allowed organisations to effectively address security threats, operational difficulties, and interruptions (Chukwu, 2024). Building next-generation AI/ML networks, improving supply chain efficiency, and increasing business efficacy will all depend on the strategic integration of AI in supply chains as we get closer to Industry 6.0 (Krishnan, 2024).

To remain competitive and innovative, organisations must have a thorough understanding of the regional differences in AI adoption, promote collaboration, and advance global AI-driven supply chain management (Atadoga, 2024).

### 2.3 Benefits and challenges in AI integration within supply chains

Supply chain management has been profoundly impacted by the development of AI technologies from Industry 4.0 to Industry 6.0. AI streamlines operations by augmenting velocity, precision, and data management skills (Modgil et al., 2021). AI applications such as robotic process automation, computer vision, and machine learning have significantly transformed decision-making and operations management in supply chains (Richey, 2023). AI-powered systems provide precise prediction of demand, optimisation of inventory, and greater visibility of the supply chain, resulting in reduced waste, cost savings, and improved ability to respond to market fluctuations (Adenekan, 2024). Using accurate demand forecasting techniques to optimise inventory levels, minimise stockouts, and reduce surplus inventory is one of the main advantages of AI integration (Belhadi et al., 2021).

Additionally, AI technologies offer significant insights into the dynamics of supply chains, predict changes in demand, and optimise inventory levels to improve customer satisfaction. Through the utilisation of artificial intelligence, organisations can acquire more profound understanding of market trends, optimise logistics operations, and improve overall efficiency and adaptability in addressing dynamic market situations (Joel, 2024). Nevertheless, there are difficulties involved with incorporating AI into supply networks. Collaboration among supply chain partners is key for improving the resilience and control of supply network risks (Belhadi et al., 2021). Supply chain managers need to evaluate their ability to efficiently incorporate AI technology (Hendriksen, 2023). It is key to thoroughly evaluate challenges in supply chain management, such as the complexity of activities, the requirement for collaboration, and the possible disadvantages linked to automation and robotics (Banur, 2024).

### 2.4 The categorical differences among Industry 4.0, 5.0, and 6.0

The development of AI in supply chain management throughout Industry 4.0, 5.0, and 6.0 represents a substantial shift in the way businesses conduct business. Industry 4.0 is defined by the pervasive implementation of automation, data analytics, and connectivity, primarily propelled by technologies including the Internet of Things (IoT), big data, and artificial intelligence (AI). During this phase, AI improves operational efficiency by facilitating real-time decision-making and predictive analytics for inventory and demand forecasting. Effective AI integration in Industry 4.0 supply chains is hampered, though, by issues including high implementation costs, worries about data security, and the requirement for new skill sets.

Industry 5.0 provides a human-centric framework, wherein AI collaborates with human labourers to develop more adaptable and tailored supply chain solutions. The emphasis transitions from mere automation to a symbiotic relationship between humans and machines, facilitating personalised services and products designed to meet individual requirements. This phase prioritises sustainability and the ethical application of AI, guaranteeing that technological development serves the interests of both enterprises and society. Organisational cultures must change to integrate human intuition with AI systems, and ethical issues like job security and human error in decision-making must also be considered.

AI manages supply chains with minimal human intervention in fully autonomous and intelligent ecosystems that Industry 6.0 envisions. During this phase, sustainability emerges as a primary objective, with AI improving not only economic efficiency but also environmental and social considerations. AI systems in Industry 6.0 are anticipated to facilitate circular economies, minimising waste, and improving resource reutilisation on a large scale. This phase, although promising, poses challenges in achieving seamless integration of autonomous systems, addressing ethical issues, and upholding governance frameworks that facilitate responsible AI implementation across interconnected supply chains.

## 3 Methodology

### 3.1 PRISMA methodology

PRISMA, or Preferred Reporting Items for Systematic Reviews and Meta-Analyses, is a widely recognised framework that offers suggestions for the conduct of systematic reviews and meta-analyses. It guarantees that research synthesis is carried out with precision, openness, and comprehensiveness. This technique is well-suited for performing a systematic evaluation on the integration of Artificial Intelligence (AI) in Supply Chain Management (SCM) from Industry 4.0 to Industry 6.0. The review will specifically focus on sustainable supply chain management. It provides a systematic method for collecting, evaluating, and summarising the existing evidence related to this study. The integration of Artificial Intelligence (AI) in supply chain management from Industry 4.0 to Industry 6.0 can be systematically analysed by researchers by adhering to the PRISMA guidelines. This methodology guarantees a thorough evaluation procedure and dependable findings. The method’s emphasis on a methodical investigation, criteria for selection, and retrieval of data is in line with the research purpose of synthesising literature on the impact of AI in various industrial revolutions on the efficiency, transparency, and resilience of supply chains. This methodology guarantees that the review may be reproduced, and the results are reliable.

The paper’s objective to comprehensively assess the role of AI in the evolution of supply chain practices is furthered by the implementation of the PRISMA approach. This method ensures a clear and direct reporting process, from the identification of research to its final inclusion, by utilising a uniform checklist and flowchart. A comprehensive approach is key for gaining a complete grasp of the extent and intricacy of ongoing research, pinpointing deficiencies, and forming solid findings that can guide future policies and regulations within the context of Industry 4.0 to Industry 6.0.

It is the most suitable option for this research due to the PRISMA framework’s systematic approach, which enables the meticulous evaluation and integration of literature. It facilitates a systematic comprehension of the transition from Industry 4.0 to Industry 6.0, providing significant observations on the influence of these technological progressions on supply chain management.

This technique offers a systematic method for assessing the consequences of adopting AI and its capacity to improve efficiency, transparency, and resilience in supply chains.

### 3.2 Inclusion and exclusion criteria

The selection criteria for literature in this systematic review are carefully crafted to provide a thorough and pertinent synthesis of the latest research on the integration of Artificial Intelligence (AI) in Supply Chain Management (SCM) from Industry 4.0 to Industry 6.0. The inclusion criteria require that studies specifically focus on the concepts, technologies, or frameworks related to Industry 4.0, Industry 5.0, and Industry 6.0. These terms indicate the progression of industrial practices and the implementation of modern technology. The literature analysed the integration of artificial intelligence (AI) in supply chain management (SCM) in certain industrial settings, with a particular emphasis on improving effectiveness, visibility, and adaptability. Studies investigating the implementation of artificial intelligence (AI) in many areas of supply chain management (SCM), including demand prediction, inventory control, transportation, and supplier management, were considered. Included in the research were discussions on the implications of AI on sustainability, encompassing environmental and social repercussions, as well as ways for attaining sustainable SCM goals. Only studies that utilised rigorous methodological approaches, such as empirical research, theoretical analysis, case studies, or systematic reviews, and were published in English from 2010 onwards, were considered. This guarantees the dependability, accuracy, and significance of the results.

The review’s focus and quality were also preserved by the establishment of specific exclusion criteria. Studies that did not specifically focus on Industry 4.0, Industry 5.0, or Industry 6.0, or did not examine the integration of artificial intelligence in supply chain management within these frameworks, were removed. Research that was not related to the industrial contexts or supply chain management (SCM) described before was considered irrelevant for this review. To prioritise recent and original research, articles published prior to 2010 or those that do not offer new insights but rather reiterate previously well-covered findings were excluded. To ensure academic rigour and credibility, non-scholarly articles, including opinion pieces, editorials, and unverified sources, were excluded. Additionally, studies that lacked adequate methodological details, rendering it impossible to evaluate their reliability and validity, were also excluded. This criterion guarantees the inclusion of only research that is well-documented and methodologically rigorous.

In addition, papers published in languages other than English were not included due to practical limitations in effectively understanding and incorporating data from non-English publications.

### 3.3 Search strategy

The search strategy for this systematic literature review was meticulously developed to identify and accumulate pertinent studies that investigated the integration of Artificial Intelligence (AI) in supply chain management from Industry 4.0 to Industry 6.0. This entailed performing a thorough and organised search across many scholarly databases to guarantee the integration of a diverse array of reliable and top-notch materials. The databases chosen for this review comprised Web of Science, Scopus, IEEE Xplore, Google Scholar, and ScienceDirect. The selection of these databases was based on their comprehensive inclusion of peer-reviewed journals, conference papers, and industry reports in the domains of industrial engineering, supply chain management, and artificial intelligence. The databases’ diversified and interdisciplinary character ensured access to a wide range of pertinent content. The selection of keywords was meticulously made and then refined through initial searches and consultations with field experts. The main keywords revolve on the terms “Industry 4.0,” “Industry 5.0,” “Industry 6.0,” “artificial intelligence,” “AI,” “supply chain management,” “digital transformation,” and “systematic literature review.” The use of Boolean operators, such as AND, OR, and NOT, was employed to proficiently merge these phrases and improve the search outcomes. For instance, searches were performed by combining phrases such as “Industry 4.0 AND artificial intelligence” and “Industry 5.0 AND supply chain management.”

In order to improve the search, the following search string was utilised: ((“Industry 4.0” OR “Fourth Industrial Revolution” OR “4IR”) AND (“Industry 5.0” OR “Fifth Industrial Revolution” OR “5IR”) AND (“Industry 6.0” OR “Sixth Industrial Revolution” OR “6IR”) AND (“artificial intelligence” OR “AI” OR “machine learning” OR “ML”) AND (“supply chain management” OR “SCM” OR “digital supply chain” OR “supply chain optimization” OR “supply chain integration” OR “smart supply chain”)). The time frame for which the search was done was January 2010–December 2023. The selected era was determined to include the latest and most important breakthroughs in the transition from Industry 4.0 to Industry 6.0. The focus is specifically on the previous decade, which has seen tremendous progress and discussions around these industrial paradigms. By restricting the search to this timeframe, the researcher guaranteed the integration of up-to-date and pertinent publications that precisely depict the present level of research and practice.

### 3.4 Data extraction and synthesis method

The systematic literature review employed a meticulously designed process that adhered to the PRISMA framework for data acquisition and synthesis. This systematic methodology ensured a thorough and clear identification, selection, and integration of relevant material that addresses the research objectives: To analyse the evolution and impact of AI technologies on supply chain management from Industry 4.0 to Industry 6.0; To identify key benefits and challenges in the integration of AI within supply chains; To provide strategic recommendations for future integration of AI in supply chains from Industry 4.0 to Industry 6.0.

A total of 34 publications that were published between 2021 and 2023 were identified during the initial search. These papers especially focus on the topic areas of supply chain management, artificial intelligence, and the progression from Industry 4.0 to 6.0. To guarantee the pertinence and excellence of the integrated research, a systematic application of inclusion and exclusion criteria was carried out. The researcher focused on publications from 2010 to 2023 there was a lack of research on the focal area of this study, hence the timeframe of 2021–2023 had to be included, due to noteworthy research being done.

The process of data extraction entailed the meticulous collection of relevant information from each of the 14 articles that were chosen. The report presented key details, including the goals, approach, notable discoveries, and the influence on supply chain management during the transition from Industry 4.0 to Industry 6.0. A standardised data extraction form was employed to ensure uniformity and comprehensiveness. This form collected key data, such as the types of AI technology discussed, the specific advantages and difficulties discovered, and any strategic suggestions for future AI integration.

The researcher employed a theme analysis approach to synthesise the data that was retrieved. This method entailed categorising the discoveries into important subjects related to the development and influence of artificial intelligence on supply chain management, spanning from Industry 4.0 to Industry 6.0. The identification of themes was accomplished through a comprehensive analysis of the literature, focussing on recurrent patterns and key ideas. These themes encompassed technology breakthroughs, integration benefits, challenges encountered, and strategic recommendations. Thematic synthesis enabled the integration of diverse data into coherent narratives that fully address the study objectives.

### 3.5 Quality assessment of the selected studies

It was imperative to conduct a quality assessment of the selected studies to guarantee the dependability and precision of the findings presented in this systematic literature review on the impact of the transition from Industry 4.0 to Industry 6.0 on sustainable supply chain management. This process entailed a thorough assessment of the methodological rigour, pertinence, and overall influence of each study on the research objectives. Studies were initially assessed and chosen based on their direct relevance to the research objectives. Only articles that only addressed subjects pertaining to Industry 4.0 and Industry 6.0, sustainable supply chain management were taken into account. The chosen publications underwent a thorough assessment of their quality using a predetermined set of criteria based on known guidelines for systematic reviews, such as the PRISMA (Preferred Reporting Items for Systematic Reviews and Meta-Analyses) framework. The evaluation criteria were centred around certain key features. Initially, the methodological rigour of each study was evaluated to guarantee strong and dependable results.

The evaluation of quantitative research was conducted by assessing their utilisation of statistical analyses, sample sizes, and control methods. The qualitative study was evaluated based on the coherence of their theoretical frameworks and the comprehensiveness of their topic analysis. The effectiveness of mixed-methods research in integrating quantitative and qualitative data to offer comprehensive insights was evaluated. Additionally, each investigation was investigated in depth to assess its significance and scope. The main criterion considered was the degree to which each study addressed the fundamental elements of Industry 4.0, Industry 6.0, sustainable supply chain management. Conducting research is necessary to obtain valuable knowledge about the impact of technological progress on supply chain, particularly in relation to sustainability and human-centred characteristics.

Furthermore, an assessment was implemented to ascertain the quality of reporting in each investigation. This entailed evaluating the lucidity and comprehensiveness of the documentation of the research procedure, the findings acquired, and the circumstances under which the research was carried out. Studies that complied with reporting requirements, which involved offering explicit explanations of their techniques, thorough discussions of their findings, and recognition of their limits, obtained superior ratings. Lastly, the influence of each study on the progression of knowledge in the areas of Industry 4.0, Industry 6.0, sustainable supply chain management was assessed.

**Figure 1:**
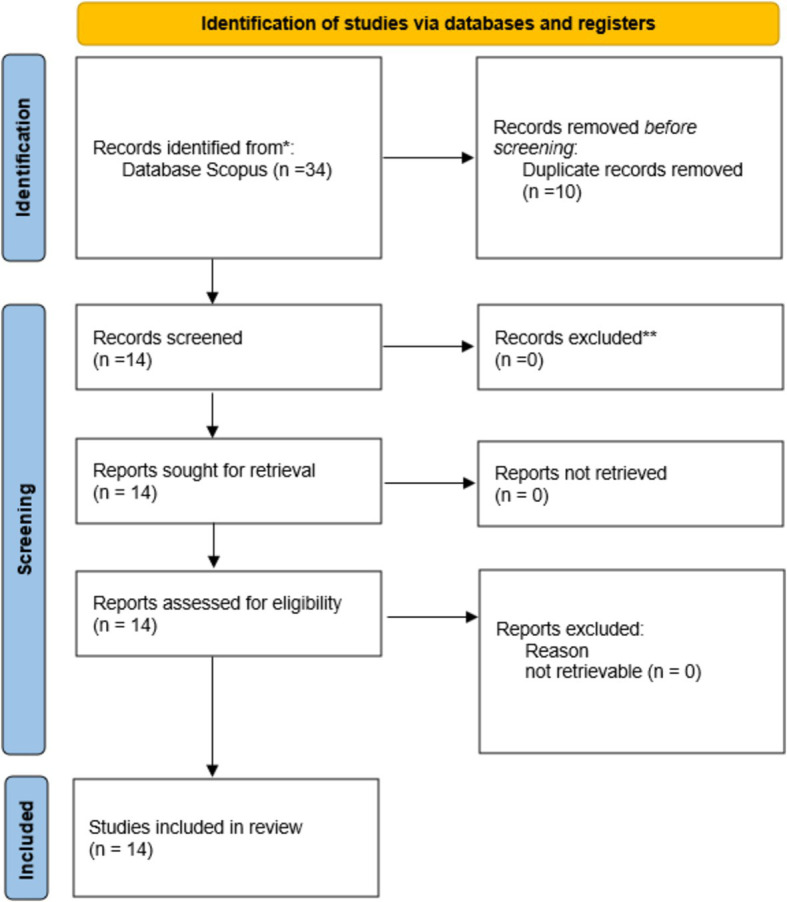
PRISMA framework. Source: Rethlefsen et al. (2021).

High-quality studies were characterised as those that offered novel viewpoints, questioned conventional paradigms, or conducted comprehensive evaluations of current literature that could inform future study and application (Figure 1).

## 4 Results

This systematic literature review investigates the integration of artificial intelligence (AI) in supply chain management (SCM) as it transitions from Industry 4.0 to Industry 6.0. The PRISMA flow diagram is used to outline the comprehensive methodology used to discover and select relevant studies. The text presents comprehensive data regarding the quantity of records discovered through database searches, the quantity of records examined, the quantity of full-text articles evaluated for suitability, and the quantity of studies incorporated in the ultimate synthesis. This rigorous methodology guarantees a methodical approach to choosing papers of excellent quality, therefore offering dependable insights into the research area (Tables 1–4).

**Table 1:** AI integration factors in supply chain management across Industries 4.0 to 6.0.

Factor	Industry 4.0	Industry 5.0	Industry 6.0
Technological Integration	IoT, Big Data, Cloud Computing	Human-Centric AI, Collaborative Robots	Advanced AI, Cyber-Physical Systems
Human-Machine Collaboration	Basic Automation	Improved Human-AI Collaboration	Seamless Human-Machine Integration
Data Management	Data Collection, Storage, and Analysis	Real-Time Data Processing, Predictive Analytics	Advanced Data Security, Ethical Data Usage
Operational Efficiency	Process Optimisation, Automated Systems	Customisation, Real-Time Decision Making	Self-Optimising Systems, Autonomous Operations
Sustainability	Initial Steps towards Sustainability	Sustainable Practices, Circular Economy	Fully Integrated Sustainable Supply Chains
Cybersecurity	Basic Cybersecurity Measures	Advanced Threat Detection, Data Privacy	Comprehensive Cybersecurity Frameworks
Skill Development	Technical Skills for Digital Tools	Training for Human-AI Collaboration	Advanced Skills for Managing AI and Sustainability
Regulatory Compliance	Adherence to Existing Industry Standards	Development of New Regulations	Strong Regulatory Frameworks for AI and Sustainability
Innovation	Incremental Innovations	Disruptive Innovations with Human-Centric Focus	Continuous Innovation Driven by AI
Customer Expectations	Improved Product and Service Quality	Personalised Products and Services	Hyper-Personalisation, Improved Customer Experience

**Table 2:** Categorical comparison of Industry 4.0, 5.0, and 6.0 with the role of AI in supply chain management.

Category	Industry 4.0	Industry 5.0	Industry 6.0	Role of AI in supply chain
Focus	Automation, digitisation, smart manufacturing	Collaboration between humans and machines	Hyper-automation, self-optimising systems	AI helps drive automation, data analysis, and decision-making across supply chain stages.
Key Technologies	IoT, Big Data, AI, robotics, cloud computing	AI, collaborative robots (cobots), human-machine interfaces	AI, quantum computing, autonomous systems	AI facilitates predictive analytics, real-time monitoring, and optimisation.
Human-Machine Interaction	Predominantly machine-driven, limited human interaction	Human-centric, with greater emphasis on personal wellbeing	Human-AI symbiosis where AI systems can autonomously learn	AI evolves from supporting decision-making to self-learning and self-improving functions.
Sustainability	Initial steps towards green manufacturing and energy efficiency	Integration of sustainability goals with human-centric innovation	Fully embedded in all processes, creating closed-loop supply chains	AI supports sustainable practices through better demand forecasting and waste reduction.
Supply Chain Management (SCM)	Focus on improving efficiency and visibility through digitalisation	Focus on resilience and flexibility, integrating human insight	Seamless, intelligent, and sustainable supply chains, fully autonomous	AI revolutionises SCM by improving flexibility, predictive analytics, and demand planning.
AI Contribution	AI aids in data-driven automation and improves productivity	AI shifts to support human decision-making and augment human roles	AI achieves full autonomy, managing dynamic and complex systems	AI improves operational efficiency, reduces errors, and facilitates decision-making.
Challenges	Data integration, high initial costs, cybersecurity	Balancing human and machine interaction, ethics, AI transparency	Managing complex, self-regulating AI systems	Complexity of integration, ethical concerns, high infrastructure requirements
Benefits of AI Integration	Improved efficiency, lower operational costs, improved production	Human-centric systems, improved creativity, personalised outcomes	Fully autonomous and intelligent systems, minimal human intervention	Increased transparency, resilience, adaptability, optimised resource allocation

**Table 3:** Summary of academic publications on human-centric technologies.

Authors	Title	Year	Cited by
Bakon K.; Holczinger T.; Sule Z.; Jasko S.; Abonyi J.	Scheduling Under Uncertainty for Industry 4.0 and 5.0	2022	17
Villar A.; Paladini S.; Buckley O.	Towards Supply Chain 5.0: Redesigning Supply Chains as Resilient, Sustainable, and Human-Centric Systems in a Post-pandemic World	2023	12
Alojaiman B.	Technological Modernizations in the Industry 5.0 Era: A Descriptive Analysis and Future Research Directions	2023	35
Borchardt M.; Pereira G.M.; Milan G.S.; Scavarda A.R.; Nogueira E.O.; Poltosi L.C.	Industry 5.0 Beyond Technology: An Analysis Through the Lens of Business and Operations Management Literature	2022	18
Ghobakhloo M.; Iranmanesh M.; Mubarak M.F.; Mubarik M.; Rejeb A.; Nilashi M.	Identifying industry 5.0 contributions to sustainable development: A strategy roadmap for delivering sustainability values	2022	155
Adel A.	Future of industry 5.0 in society: human-centric solutions, challenges and prospective research areas	2022	258
Barata J.; Kayser I.	Industry 5.0 - past, present, and near future	2023	39
Nozari H.	Supply chain 6.0 and moving towards hyper-intelligent processes	2023	7
Saniuk S.; Grabowska S.; Straka M.	Identification of Social and Economic Expectations: Contextual Reasons for the Transformation Process of Industry 4.0 into the Industry 5.0 Concept	2022	109
Yesodha K.R.K.; Jagadeesan A.; Gowrishankar V.; Logeshwaran J.	IOT enabled real time data exchange to resolve bottlenecks and streamline workflow in factories	2023	6
Patnaik P.; Nayak P.; Misra S.	Personalized Product Recommendation and User Satisfaction: Reference to Industry 5.0	2023	0
Kalateh S.; Estrada-Jimenez L.A.; Pulikottil T.; Hojjati S.N.; Barata J.	Feeling Smart Industry	2021	2
Jefroy N.; Azarian M.; Yu H.	Moving from Industry 4.0 to Industry 5.0: What Are the Implications for Smart Logistics?	2022	84
Pyun J.; Rha J.S.	Review of research on digital supply chain management using network text analysis	2021	17

**Table 4:** Main themes, sub themes and supporting quotes.

Main theme	Sub theme	Supporting quotes
Technological Developments	Industry 4.0	Bakon et al. (2022): “Managing uncertainties in Industry 4.0 environments through advanced technologies…” Ghobakhloo et al. (2022): “Digitalisation plays a pivotal role in Industry 4.0….” Borchardt et al. (2022): “Industry 4.0 improves supply chain efficiency….”
	Industry 5.0	Villar et al. (2023): “Supply Chain 5.0 integrates human-centric technologies…” Alojaiman (2023): “Industry 5.0 focuses on human-computer interaction…” Borchardt et al. (2022): “Human-robot collaboration is central to Industry 5.0…”
	Digitalisation	Ghobakhloo et al. (2022): “Digitalisation is key for sustainable production…” Villar et al. (2023): “Digitalisation improves supply chain responsiveness…” Bakon et al. (2022): “Digitalisation supports decision-making in Industry 4.0…”
Human-Centric Approaches	Human-robot collaboration	Borchardt et al. (2022): “Human-robot collaboration is key in Industry 5.0….” Alojaiman (2023): ‘Robots and humans work together in modern industries…” Villar et al. (2023): “Collaboration between robots and humans boosts efficiency…”
	Human-computer interaction	Alojaiman (2023): “Human-computer interaction is vital in Industry 5.0….” Borchardt et al. (2022): “Industry 5.0 emphasizes human-computer collaboration…” Villar et al. (2023): “Improved human-computer interaction improves productivity…”
	Collaborative manufacturing	Villar et al. (2023): “Collaborative manufacturing integrates human-centric technologies…” Borchardt et al. (2022): “Collaborative approaches improve manufacturing efficiency…” Alojaiman (2023): “Collaboration in manufacturing is key for Industry 5.0…”
Sustainability	Environmental sustainability	Ghobakhloo et al. (2022): “Industry 5.0 supports environmental sustainability…” Villar et al. (2023): “Sustainability is integrated into modern supply chains…” Borchardt et al. (2022): ‘Environmental sustainability is imperative for future industries…”
	Sustainable production	Ghobakhloo et al. (2022): “Sustainable production is a focus in Industry 5.0…” Bakon et al. (2022): “Sustainability drives modern production processes…” Alojaiman (2023): “Sustainable practices are key for Industry 5.0…”
	Supply chains	Bakon et al. (2022): “Supply chains benefit from Industry 4.0 technologies…” Villar et al. (2023): “Supply Chain 5.0 incorporates sustainable practices…” Ghobakhloo et al. (2022): “Sustainable supply chains are key for modern industries…”

**Figure 2:**
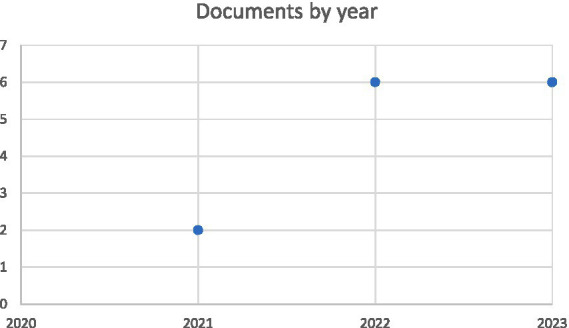
Documents by year.

The line graph in Figure 2 provides a comprehensive data visualisation that successfully communicates the extensive research conducted in the field of human-centric technologies from 2021 to 2023. The line graph depicts the sequential progression of academic publications, with the x-axis representing the years and the y-axis specifying the annual count of documents generated. This visual depiction enables a quick and thorough understanding of patterns and fluctuations in research activity within the chosen period. In this perspective, the graph shows that only two documents were produced in 2021, indicating a relatively low volume of research in the area of human-centric technology during this particular year. In 2022, the quantity underwent a tripling, as demonstrated by the publication of six documents. This signifies a significant increase in both interest and scholarly productivity in this area of study.

However, the most significant stability was observed in 2023, when the number of documents remained consistent at six. The steady creation of academic research in human-centric technology indicates a long-term interest and ongoing efforts in this field. The prominent expansion projected for 2022 and consistent output anticipated for 2023 may indicate an increasing recognition of the importance of human-centric technologies, potentially driven by global environmental initiatives and improved funding opportunities in this domain. Conducting an analysis of the articles from a particular time frame may disclose trends in the academic focus of that period. For instance, the restricted output in 2021 may suggest initial exploratory studies or foundational research that is laying the groundwork for future extensive investigations. The large increase in 2022 suggests a shift towards broader research or the use of emerging technologies and methodologies in human-centric technologies. Continued dedication to the exploration and development of the foundation established in previous years is evidenced by the consistent output in 2023.

**Figure 3:**
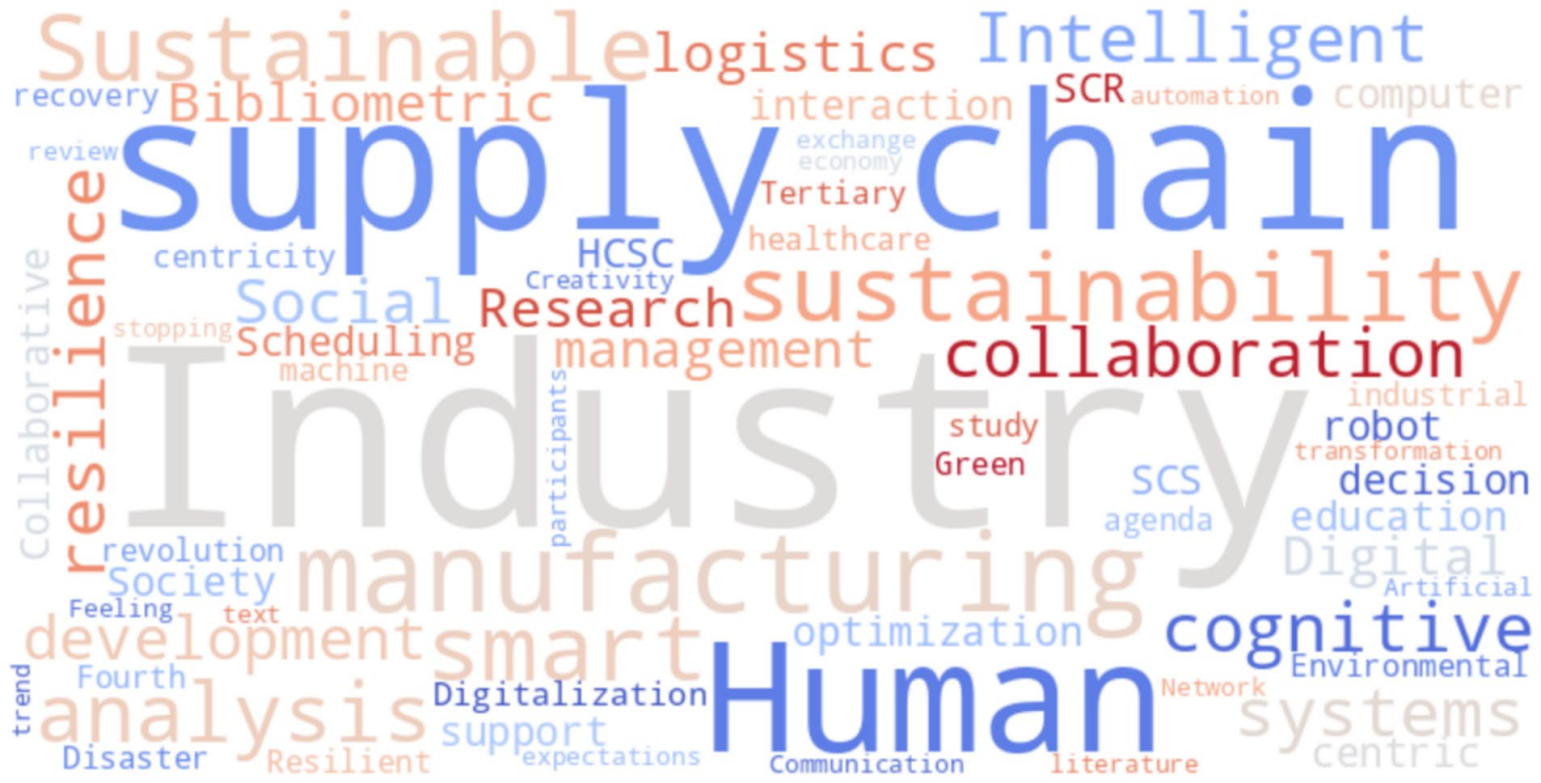
Documents by type.

The data visualisation featured in Figure 3, “Document types,” offers a comprehensive overview of the research conducted in the field of human-centric technologies, arranged according to document categories. The pie chart provides a concise representation of the distribution of several forms of academic publications, highlighting the predominant formats used by scholars in this field. According to the chart, review articles are the most prevalent document form, with a total of five publications. This indicates that there is a significant body of meticulous research on human-centric technology being documented in review articles, which signifies a need for thorough investigations of the topic that are suitable for academic and professional use.

Four publications are presented below, each of which exhibits a significant quantity of research sharing through articles. This statement emphasises the ongoing academic discourse and the importance of human-centred technology as a topic of scholarly investigation within the broader academic community. Conference papers are distinct publications that highlight participation at academic conferences, where researchers share and discuss their research findings. The inclusion of human-centric technologies subjects in larger edited volumes is suggested by the fact that each book chapter represents two independent publications. This suggests a method of studying that involves working together, with individuals making contributions within the context of larger enquiries on specific topics.

Examining the papers over time can disclose patterns in the academic focus over the selected period, enabling us to ascertain whether there is a change in attention across various disciplines of study. An examination of the trajectory of review articles, articles, conference papers, and book chapters creation over time could offer valuable insights into shifts in research objectives or the emergence of novel areas of focus in human-centric technologies. Conducting such an analysis is critical for identifying changing priorities and deficiencies in the existing literature, which may uncover areas that need further investigation.

The table offers an insightful summary of academic publications focused on human-centric technologies, detailing the authors, titles, years of publication, and citation counts. This information highlights the scope and influence of research within this domain over recent years. Publications from 2022 and 2023 dominate the table, reflecting a growing interest in human-centric technologies. For instance, “Scheduling Under Uncertainty for Industry 4.0 Using Multi-Agent Systems” by Bakon et al. (2022) published in 2022, has been cited 17 times, indicating significant interest in managing uncertainties within Industry 4.0 environments through advanced technologies.

“Towards Supply Chain 5.0: Redesigning Supply Chains with Human-Centric Technologies” by Villar et al. (2023) from 2023, has 12 citations, showcasing the evolving nature of supply chains and the integration of human-centric technologies to improve efficiency and responsiveness. Similarly, “Technological Modernizations in the Industry 5.0 Era: A Focus on Human-Computer Interaction” by Alojaiman (2023), also from 2023, with 35 citations, highlights the growing interest in developments within human-computer interaction in Industry 5.0. An important contribution from 2022, “Industry 5.0 Beyond Technology: An Analysis Through the Lens of Human-Robot Collaboration” by Borchardt et al. (2022) has gathered 18 citations. This publication underlines the significance of human-robot collaboration in Industry 5.0, an area of increasing importance in both academic and practical contexts.

The most cited work, “Identifying Industry 5.0 Contributions to Sustainable Production” by Ghobakhloo et al. (2022) published in 2022 with 155 citations, emphasises the pivotal role of Industry 5.0 in sustainable production. This high citation count reflects the significant impact and recognition of research focused on integrating sustainability with technological developments.

### 4.1 Thematic analysis

**Figure 4:**
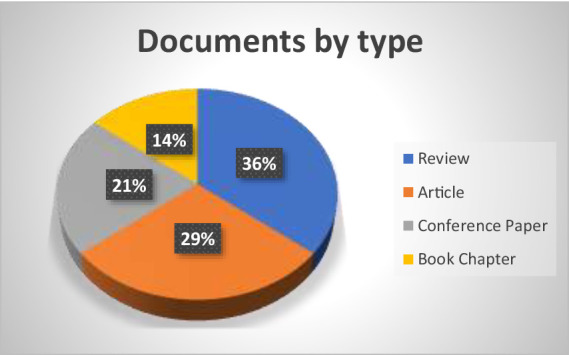
Word cloud.

Figure 4, provides a visual representation of the most frequently occurring terms in the research field is provided by the word cloud generated from the keywords related to human-centric technologies. The visible display of these keywords in different sizes and colours facilitates the rapid identification of the main areas of concentration and the relative significance of different concepts within the domain. The central function of recent research is indicated by the prominent inclusion of terms such as “Industry 4.0” and “Industry 5.0” in the word cloud. These phrases represent the considerable focus on technical progress and shifts from Industry 4.0, which emphasises automation and data interchange, to Industry 5.0, which combines human-centred approaches and collaboration with modern technologies.

Additional important terms include “Sustainability,” “Human-robot collaboration,” and “Digitalisation.” The significance of “Digitalisation” emphasises the extensive implementation and integration of digital technologies in diverse industrial and organisational procedures. This indicates an increasing acknowledgement of the significance of digital transformation in attaining effectiveness and novelty. The terms “Human-computer interaction” and “Human-robot collaboration” are also profoundly highlighted, emphasising the growing emphasis on collaborative technologies that improve human-machine connection. This signifies a transition towards technologically advanced solutions that are more integrated and focused on human needs. It involves utilising the combined power of human abilities with advanced robotics or computer systems to improve productivity and achieve better results.

The word cloud created from the keywords in human-centric technologies research provides a clear visual summary of the dominant themes and their relative importance in the field. Key terms such as “Industry 4.0” and “Industry 5.0” appear prominently, indicating a significant focus on these technological developments. These terms highlight the transition from automated, data-driven environments to more human-centric, collaborative systems.

Digitalisation also stands out as a critical theme, reflecting its key role in enabling advanced technological solutions and improving efficiency and responsiveness in various processes. The emphasis on “Human-robot collaboration” and “Human-computer interaction” underlines the importance of integrating human skills with technological capabilities, a central aspect of Industry 5.0.

**Figure 5:**
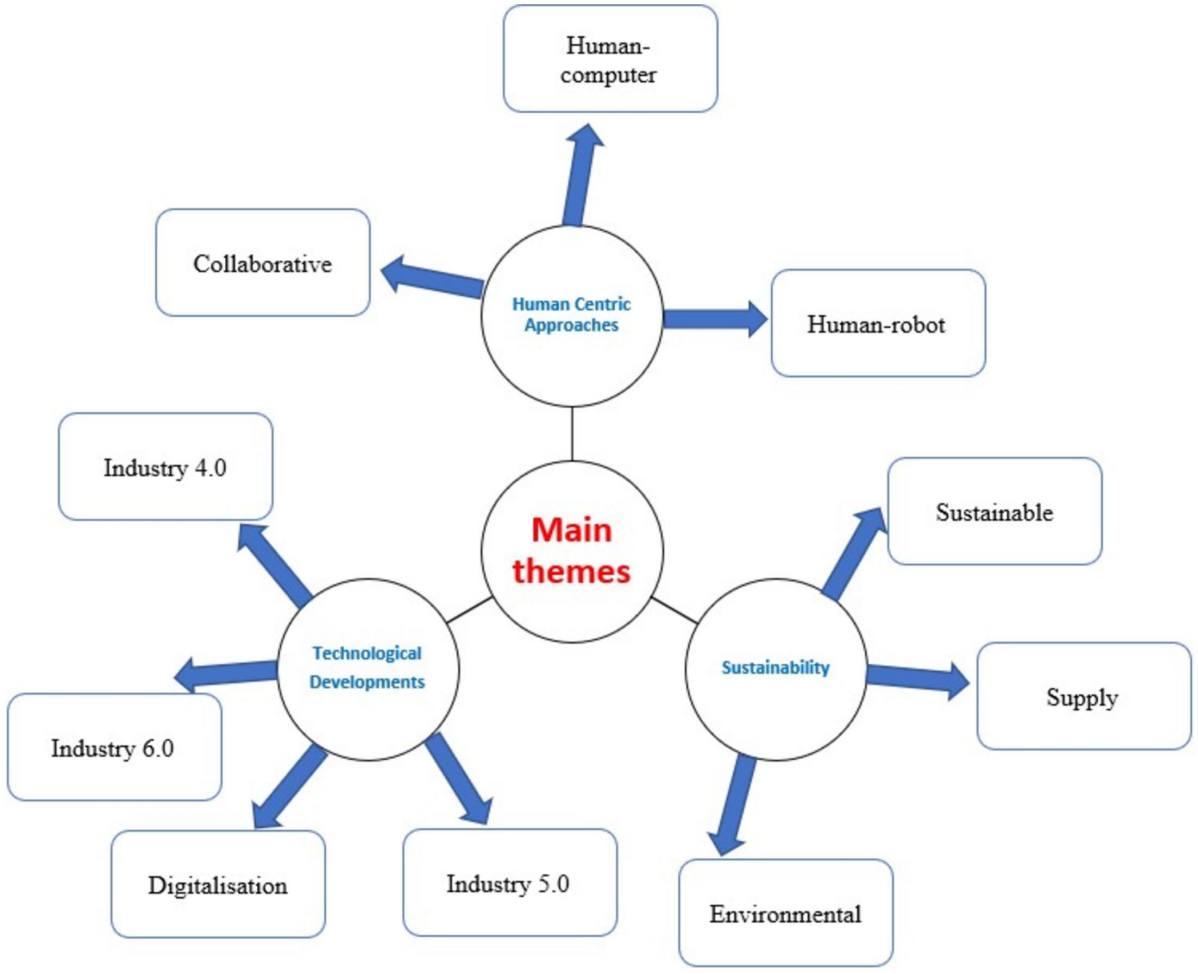
Main themes, subthemes.

Sustainability is another major theme, with frequent mentions of “Environmental sustainability,” “Sustainable production,” and “Supply chains.” This indicates a strong commitment to incorporating sustainable practices in technological developments, ensuring that environmental considerations are integral to the development of new technologies and industrial processes. The word cloud effectively highlights these core themes, providing a concise overview of the research focus on human-centric technologies (Figure 5).

## 5 Discussion

### 5.1 Theme one: technological developments

In the context of analysing the integration of Artificial Intelligence (AI) in Supply Chain Management from Industry 4.0 to 6.0, it is imperative to evaluate the evolution and influence of AI technologies on supply chains, identify the primary benefits and challenges associated with AI integration, and offer strategic recommendations for future integration. Industry 4.0 signifies the contemporary stage of the industrial revolution, marked by the integration of digital technologies into production and logistics operations. This stage focusses on the implementation of automation, data interchange, and intelligent technologies to improve efficiency and production (Tahera et al., 2023). In the future, Industry 5.0 is anticipated to be a human-centric approach that seeks to balance the roles of humans and machines in the production process.

The emphasis is on encouraging collaboration between humans and AI systems to improve decision-making and problem-solving processes. The primary focus is on ensuring the wellbeing of workers and tailoring products to match individual demands (Tahera et al., 2023).

Real-time data sharing, predictive analytics, and process automation are all facilitated by digitalisation, which is key for the transformation of supply chain management. The process of digitalisation improves the level of visibility, transparency, and agility in supply chains, resulting in better decision-making and operational efficiency (Reis-Andersson, 2023). Artificial intelligence (AI) may generate numerous subthemes when integrated into supply chains. These features may encompass the implementation of AI technologies, including data-driven decision-making, predictive analytics for forecasting demand, autonomous systems for managing inventories, and AI-driven optimisation of logistics and transportation processes. Challenges may encompass concerns such as data security and privacy, the requirement to improve the skills of the workforce to effectively engage with AI systems, and the assurance of ethical use of AI across the supply chain (Eyo-Udo, 2024).

It is imperative to evaluate the evolving role of AI in improving the resilience, sustainability, and customer contentment of supply chains as they transition from Industry 4.0 to 6.0 in order to formulate strategic recommendations for future AI integration. Suggestions may centre around allocating resources towards the development of AI expertise, promoting cooperation between AI systems and human employees, and implementing strong governance structures to enable responsible deployment of AI in supply chain operations (Tahera et al., 2023).

### 5.2 Theme two: human-centric approaches

In the transition from Industry 4.0 to Industry 6.0, human-centric approaches to the integration of artificial intelligence (AI) in supply chain management encompass a variety of subthemes, including human-robot collaboration, human-computer interaction, and collaborative manufacturing. Human-robot collaboration (HRC) refers to the efficient cooperation between humans and machines to accomplish activities (Zhao et al., 2021). This collaboration is key in smart manufacturing environments to capitalise on the capabilities of robotic systems and human experience (Othman and Yang, 2022). HRC, or Human-Robot Collaboration, improves productivity and output quality in manufacturing processes by combining the flexibility of humans with the efficiency of robots (Liu and Wang, 2021).

Nevertheless, despite the potential advantages, the industry continues to show reluctance in completely adopting HRC (Rega et al., 2021). Within the field of human-computer interaction, context awareness is used to guarantee human-robot collaboration without collisions (Liu et al., 2021). This form of contact is key for improving safety and efficiency in manufacturing operations.

Furthermore, there is a growing recognition of the importance of adopting designs that utilise depth sensor-based hand gesture communication for human-robot interaction in smart material-handling robot operations (Ding and Su, 2022). The progress in human-robot interaction is set to transform car manufacturing and improve operational efficiency. The transition from Industry 4.0 to Industry 6.0 is largely dependent on collaborative manufacturing, which integrates resources from many businesses to maximise production capacity (Tang et al., 2023). By utilising collective resources, organisations may effectively respond to the competitive market scenario, resulting in increased productivity and innovation. However, collaborative manufacturing is complicatedly connected to the establishment of supply chain collaborations within the Industry 4.0 framework (Cisneros-Cabrera et al., 2021). In this context, effective collaborations require the rapid formation of a team, the selection of a diverse array of suppliers, the establishment of trust among members, and the ability to scale to accommodate dynamic production demands.

### 5.3 Theme three: sustainability

Environmental sustainability, sustainable production, and sustainable supply chains are key subthemes to consider when analysing the integration of Artificial Intelligence (AI) in supply chain management from Industry 4.0 to 6.0. The importance of sustainable supply chain management in contemporary company operations is widely acknowledged due to escalating environmental concerns and the imperative for sustainable development (Chen, 2024). This entails the thorough administration of resources, reduction of detrimental environmental impacts, and development of social fairness and economic sustainability (Abaku, 2024). Sustainable supply chains have a significant impact in reducing the adverse environmental effects of organisations, including greenhouse gas emissions, resource wastage, and harm to ecosystems (Li, 2023).

Environmental sustainability is a critical component of sustainable supply chain practices, which encompasses the reduction of waste and emissions, the utilisation of renewable energy, the sustainable sourcing of materials, and the promotion of equitable labour practices (Hasrul, 2023). It is key to incorporate environmental sustainability throughout every stage of the global value chain to optimise resource utilisation and reduce environmental harm (Zhang, 2023). Additionally, sustainable supply chain development encompasses multiple facets of supply chain management, such as development, design, production, packaging, marketing, distribution, consumption, and recycling.

**Figure 6:**
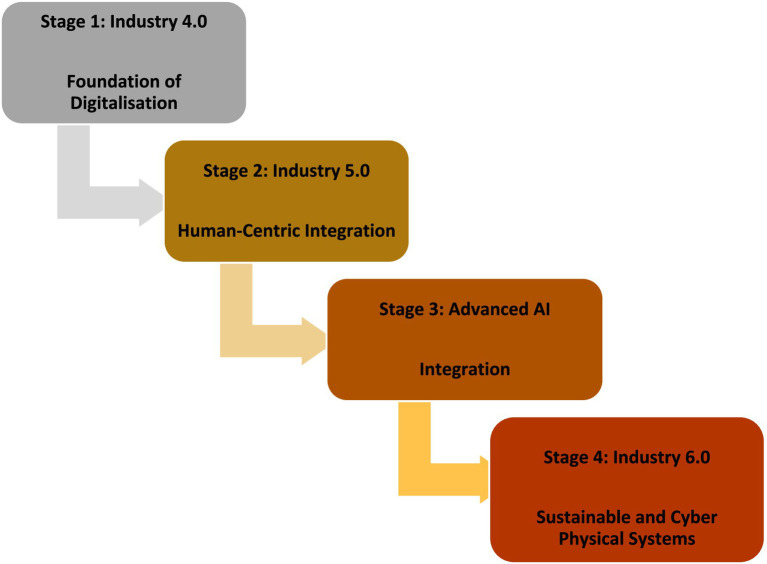
Stages of Industry Evolution from Industry 4.0 to Industry 6.0.

The objective is to minimise negative environmental effects while attaining sustainability objectives (Qu and Ji, 2023). Okoye (2024) emphasises the importance of minimising environmental impacts during the production process in the context of sustainable production. This can be achieved by implementing eco-friendly logistics strategies, utilising renewable energy in transportation and distribution networks, and emphasising responsible sourcing. Adopting sustainable production processes is key for attaining sustainability objectives and minimising the environmental impact of supply chains (Reynolds, 2024). However, sustainable supply chain management entails incorporating triple bottom line objectives (environmental, social, and economic) to improve long-term economic performance and encourage sustainable development (Mursidah and Fauzi, 2022) (Figure 6).

### 5.4 Recommended proposed model: stages of industry evolution

#### 5.4.1 Stage one: foundation of digitalisation

The industrial sector’s digitisation is based on the first stage, known as Industry 4.0. This phase is distinguished by the integration of digital technologies into production procedures, resulting in the establishment of intelligent factories. Key technologies include cloud computing, cyber-physical systems, big data analytics, and the Internet of Things (IoT). These technologies provide the continuous monitoring of systems, the ability to anticipate and prevent maintenance issues, and the use of data to inform decision-making, resulting in a significant improvement in operational efficiency and productivity. Industry 4.0 revolutionises conventional manufacturing through the promotion of connectivity and automation, which sets the foundation for future improvements in industrial development.

#### 5.4.2 Stage two: human-centric integration

Industry 5.0 emphasises human-centric integration, building upon the digital foundation of Industry 4.0. This stage emphasises the synergy between humans and technology, where cutting-edge technologies improve human abilities instead of substituting them. Key components comprise of collaborative robots (cobots), artificial intelligence (AI), and sophisticated human-machine interfaces. Industry 5.0 pursues to establish a mutually beneficial connection between workers and intelligent systems, amplifying creativity, problem-solving, and customisation in production procedures. This stage focusses on the promotion of human wellbeing, ensuring that technology progress contributes to a more sustainable and inclusive industrial setting.

#### 5.4.3 Stage 3: AI advanced integration

In the third step, known as Advanced AI Integration, industrial processes are deeply integrated with artificial intelligence. This level employs machine learning, deep learning, and advanced analytics to optimise processes, increase decision-making, and improve predictive capabilities. AI-powered systems have the capability to assess large volumes of data in real-time, detecting patterns, forecasting results, and independently making decisions to improve productivity and minimise operational interruptions. Integrating advanced AI technology allows for the creation of intelligent supply chains, which improve responsiveness and adaptability through end-to-end visibility and automation. This stage signifies a significant development towards independent and self-improving industrial systems.

#### 5.4.4 Stage 4: sustainable and cyber physical systems

Industry 6.0 is the culmination of industrial evolution, emphasising the integration of cyber-physical systems and sustainability. The objective of this stage is to establish manufacturing ecosystems that are both strong and sustainable, achieved through the integration of advanced digital technology and sustainable practices. Cyber-physical systems, which combine physical processes with computing components, are key for accomplishing this objective. They provide the continuous monitoring, regulation, and improvement of industrial processes in real-time, resulting in heightened effectiveness and diminished ecological footprint.

Industry 6.0 prioritises the application of circular economy ideas, renewable energy sources, and resource efficiency to ensure that industrial expansion is in harmony with environmental sustainability and social responsibility.

### 5.5 Theoretical and practical implications

The theoretical implications of the integration of Artificial Intelligence (AI) into supply chain management from Industry 4.0 to Industry 6.0 are profound, as it improves our comprehension of technological evolution and its influence on operational efficiency. In theory, the results of the systematic literature study emphasise the revolutionary capacity of AI technology in optimising supply chain processes. The use of AI in predictive analytics, demand forecasting, and decision-making is consistent with established theories on technological progress and operational effectiveness. The shift from Industry 4.0, marked using digital technology, to Industry 6.0, which prioritises sustainability and human-centred integration, aligns with theoretical frameworks that promote ongoing technical development and its alignment with goals for sustainable development.

The literature emphasises the significance of integrating AI into supply chains to improve responsiveness, resilience, and agility. This is consistent with the resource-based view (RBV) of competitive advantage, which suggests that companies achieve a competitive advantage by strategically utilising resources, such as superior technologies. Additionally, the integration of AI aligns with the dynamic capability’s hypothesis, which highlights the need for organisations to adjust, incorporate, and reorganise their internal and external skills to effectively respond to quickly evolving surroundings. This research makes theoretical advances by strengthening current theories and offering empirical proof of the influence of AI on supply chain management.

The results provide practical insights for businesses that are seeking to improve their supply chain operations by integrating AI. The practical implications are significant as they offer a clear plan for organisations to efficiently integrate AI technologies. The shift from Industry 4.0 to Industry 6.0 requires a planned method of incorporating AI, with a focus on creating systems that prioritise human needs and talents, rather than replacing them. This method guarantees that technological developments improve both the overall efficiency of operations and the wellbeing of workers. One of the primary practical implications is the necessity of strong data management systems to facilitate AI applications.

Organisations should allocate resources to implement sophisticated data analytics and machine learning algorithms to fully leverage the capabilities of artificial intelligence in predictive maintenance, demand forecasting, and inventory optimisation.

However, the integration of AI technology might result in significant cost savings and improved efficiency, especially in the fields of logistics and transportation. Through the automation of repetitive processes and the optimisation of transportation routes, artificial intelligence (AI) has the potential to decrease operational expenses and improve delivery efficiency. The significance of addressing cybersecurity and data privacy challenges is also underlined by the research. With the increasing interconnectivity and dependence on digital technologies, it is key to prioritise the protection of sensitive information in supply chains. Practical suggestions involve the implementation of thorough cybersecurity measures and the establishment of legal frameworks to guarantee the ethical use of AI in supply chain management. Additionally, the emphasis on sustainability in Industry 6.0 implies that organisations should improve their supply chain operations by prioritising sustainable practices. This entails embracing the ideas of circular economy, employing renewable energy sources, and minimising waste. By combining artificial intelligence with sustainable practices, firms may attain both operational efficiency and fulfil their social and environmental responsibilities. This will ultimately improve their company reputation and ensure compliance with regulatory standards.

### 5.6 Limitations of the study

Initially, by restricting the scope of the literature to works published between 2010 and 2023, papers considered are only from the last 3 years of the period 2010–2023. Noteworthy older publications that could offer invaluable perspectives on the development of artificial intelligence in supply chain management may have been overlooked. Furthermore, the dependence on English-language publications may lead to a linguistic prejudice, potentially disregarding relevant research carried out in other languages. However, the rapid growth in AI and supply chain management technology implies that certain discoveries may rapidly become obsolete. Due to its narrow focus on industrial settings, the study’s breadth might not have adequately captured the wide range of uses and complications of AI in all industries. The integration of AI into supply chains is a developing area, and the existing research may not yet cover all the practical difficulties and complexities.

## 6 Conclusion and recommendations

The integration of Artificial Intelligence (AI) has yielded pivotal insights, as supply chain management transitions from Industry 4.0 to Industry 6.0. AI improves operational efficiency and decision-making by leveraging sophisticated analytics and machine learning algorithms, resulting in better demand forecasting and inventory control. Industry 5.0 notably emphasises a human-centric paradigm, promoting collaboration between humans and AI systems to improve customisation and problem-solving abilities. Through resource optimisation and environmental impact reduction, this synergy not only increases operational efficacy but also advances sustainability, supporting Industry 6.0’s objectives. Nevertheless, these advantages, challenges including cybersecurity vulnerabilities and the necessity for workforce upskilling remain.

The development of systems that are sustainable, efficient, and resilient is the primary focus of strategic recommendations for the future integration of AI into supply chains. Organisations ought to invest in sophisticated AI technologies to improve competencies in real-time data analysis, predictive analytics, and decision-making. This investment should include using machine learning and deep learning techniques to improve supply chain operations and forecast market trends. Equally key is the implementation of stringent cybersecurity protocols to safeguard supply chain data from cyber threats, thereby ensuring the integrity and security of AI-improved systems.

Additionally, a human-centric integration is key, in which AI systems complement rather than supplant human capabilities. This entails providing employees with the requisite skills and knowledge for efficient collaboration with AI, encouraging an environment that promotes productivity and innovation. Sustainability must be prioritised, with AI applications designed to improve environmentally friendly supply chain practices, including minimising carbon footprints, optimising resource utilisation, and advancing circular economy models. However, strong regulatory frameworks are crucial for overseeing the ethical application of AI in supply chains, tackling concerns regarding privacy, transparency, and equity. Continuous improvement and innovation are key, and they can be achieved by encouraging collaboration among industry stakeholders, academic institutions, and technology providers to keep up with technological developments and emerging trends.

These strategies will enable a smooth transition from Industry 4.0 to Industry 6.0, establishing resilient, flexible, and eco-friendly supply chains.

Future studies about supply chain management and AI integration should focus on technological, human-centred, and sustainable issues. Investigating the cross-industry integration of artificial intelligence (AI) in supply chain management uncovers significant deficiencies in standardisation and regulatory adaptation among sectors like healthcare, agriculture, and retail. The differing adoption rates and regulatory frameworks among these industries highlight the necessity for standardised AI protocols that facilitate seamless interoperability while accommodating distinct operational and compliance requirements. A comprehensive regulatory framework could promote improved predictive analytics, data sharing, and optimisation capabilities that are versatile across industries, thereby facilitating more robust and efficient supply chains. However, the establishment of cross-industry AI standards would improve ethical considerations, including data privacy and transparency, which are especially critical in sensitive sectors where consumer trust is important. This approach improves operational efficiency and aligns with new regulatory expectations, positioning AI as a strategic facilitator for innovation and compliance across various industrial applications.

Empirical research is key to assess the enduring effects of AI on supply chain resilience and efficiency across diverse sectors. These studies should evaluate both the advantages and challenges related to AI technologies such as machine learning and predictive analytics in improving supply chain operations. Research must also investigate the human-centric dimensions of AI integration, specifically the improvement of collaborative robotics (cobots) and sophisticated human-machine interfaces to augment human capabilities. Furthermore, it is critical to investigate how AI can be used to advance sustainable supply chain practices and to develop rigorous frameworks for assessing the social and ecological effects of supply networks driven by AI. It is key to address cybersecurity and data privacy in AI-integrated supply chains, needing the establishment of thorough regulatory frameworks and best practices to protect sensitive data and promote trust and acceptance within the industry.
